# The new child food package is associated with reduced obesity risk among formula fed infants participating in the Special Supplemental Nutrition Program for Women, Infants and Children (WIC) in Los Angeles County, California, 2003–2016

**DOI:** 10.1186/s12966-020-0921-3

**Published:** 2020-02-10

**Authors:** M. Pia Chaparro, Christopher E. Anderson, Catherine M. Crespi, May C. Wang, Shannon E. Whaley

**Affiliations:** 1 Department of Global Community Health and Behavioral Sciences, School of Public Health and Tropical Medicine, Tulane University, 1440 Canal St., suite 2200-16, mail code #8319, New Orleans, LA 70112 USA; 2 Department of Epidemiology, School of Public Health and Tropical Medicine, Tulane University, 1440 Canal St., suite 2000, New Orleans, LA 70112 USA; 3 Public Health Foundation Enterprises (PHFE) WIC, 12781 Schabarum Ave, Irwindale, CA 91706 USA; 4 Department of Biostatistics, Fielding School of Public Health, University of California Los Angeles, 650 Charles E. Young Drive Dr. South, Box 951772, Los Angeles, CA 90095 USA; 5 Department of Community Health Sciences, Fielding School of Public Health, University of California Los Angeles, 650 Charles E. Young Drive Dr. South, 26-051B CHS, Los Angeles, CA 90095 USA

**Keywords:** WIC, Obesity, Formula feeding, Los Angeles County

## Abstract

**Background:**

The Special Supplemental Nutrition Program for Women, Infants and Children (WIC) changed the food packages provided to its participants in 2009, to better align them with the Dietary Guidelines for Americans. Previous research found that the 2009 WIC food package change was associated with reduced obesity risk, particularly among breastfed infants but also among those who were never breastfed. The objective of this study was to determine if the new *child* food package introduced in 2009, including more produce and whole grains for 1–4-year old children, was associated with healthier growth trajectories and reduced obesity risk at age 4 years among children who were exclusively formula fed during infancy.

**Methods:**

Administrative data on WIC-participating children in Los Angeles County, 2003–2016, were used (*N* = 74,871), including repeated measures of weight and length (or height); child’s age, gender, and race/ethnicity; maternal education and language; and family poverty. Gender-stratified spline mixed models were used to examine weight-for-height z-score (WHZ) growth trajectories from 0 to 4 years and Poisson regression models were used to assess obesity (BMI-for-age > 95th percentile) at age 4. The main independent variable was duration of receipt (dose) of the new child package, categorized as 0, > 0 to < 1, 1 to < 2, 2 to < 3, 3 to < 4, and 4 years.

**Results:**

WHZ growth trajectories were similar for children across new child package dose groups. Boys and girls who were fully formula fed during infancy but received the new *child* food package for 4 years had a 7% (RR = 0.93; 95%CI = 0.89–0.98) and a 6% (RR = 0.94; 95%CI = 0.89–0.99) lower obesity risk, respectively, compared to children who received the new *child* food package for 0 years. There were no differences in obesity risk for children receiving < 4 years of the new *child* package vs. 0 years.

**Conclusions:**

Providing healthy foods during childhood to children who were exclusively formula fed as infants was associated with modest improvements in obesity outcomes. While breastfeeding promotion should still be prioritized among WIC participants, providing healthy foods during childhood may provide health benefits to formula fed children, who comprise a sizeable proportion of children served by WIC.

## Background

The Special Supplemental Nutrition Program for Women, Infants and Children (WIC) is a federal nutrition assistance program in the United States, serving pregnant, lactating, and postpartum women as well as infants and children up to the age of 5 years, who live in low-income households and are at nutritional risk. WIC provides supplemental foods, nutrition education, breastfeeding support, and medical and social service referrals to its participants. In 2018, 6.9 million families were served by the WIC program each month, including approximately half of all infants born in the USA, for a total annual cost of US$5.3 billion [1].

In 2009, WIC updated the food packages their beneficiaries receive to better align them with federal dietary guidelines [2]. These changes included the addition of fruits, vegetables, and whole grains; a reduction in the amount of dairy, juice, and eggs; and a calibration in formula amounts to match infants’ age and needs [2]. One of the objectives of the new WIC food package was to incentivize breastfeeding; this was to be accomplished by expanding benefits, both in terms of quantity and variety, to mothers who chose to exclusively breastfeed for longer and also to their infants ages 6–11.9 months [2, 3]. Previous studies show that this objective was successful: in Los Angeles County (California), for example, the uptake of the *fully breastfeeding* package among women increased by 86% after the food package change [4]. A study including 17 local WIC agencies across 10 U.S. states also found an increase in the issuance of the *fully breastfeeding* package post-2009, from 9.8 to 17.1%, a 74% increase [5]. However, the latter study also found an increase in the issuance of the *fully formula feeding* package from 20.5 to 28.5%, with an accompanied reduction in mixed feeding [5]. In fact, children who consume some formula remain a majority among children served by WIC, particularly after the first month of life [5].

We recently found that children who were enrolled in WIC in Los Angeles County continuously from birth until age 4 (inclusive) and who received the new food package, compared to the old, had a 10–12% lower obesity risk at age 4 [6]. Further, we found that an increase in the amount of breastfeeding was partially responsible for the positive effect of the new food package change on obesity [7]. However, we also observed a reduced obesity risk among children who participated in WIC post-2009 and received the *fully formula feeding* package from 0 to 12 months of age (i.e., never breastfed), compared to fully formula feeders participating in WIC pre-2009 [7]. This finding implies that the food package changes may have been beneficial beyond their effect on breastfeeding duration, suggesting that the new *child* package provided between 1 and 4 years and/or other changes in the infant package (the calibration of infant formula amounts from 0 to 12 months or the elimination of juice and the addition of baby food fruits and vegetables from 6 to 11.9 months) may have been responsible for the reduced obesity risk observed among formula fed infants [6]. Therefore, the aims of this study were to investigate 1) the effect of duration of receipt (dose) of the new *child* food package; and 2) the effect of the new *infant* food package on growth trajectories from 0 to 4 years and obesity risk at age 4 among children who participated in WIC in Los Angeles County between 2003 and 2016 and who were fully formula fed as infants.

## Methods

**Fig. 1:**
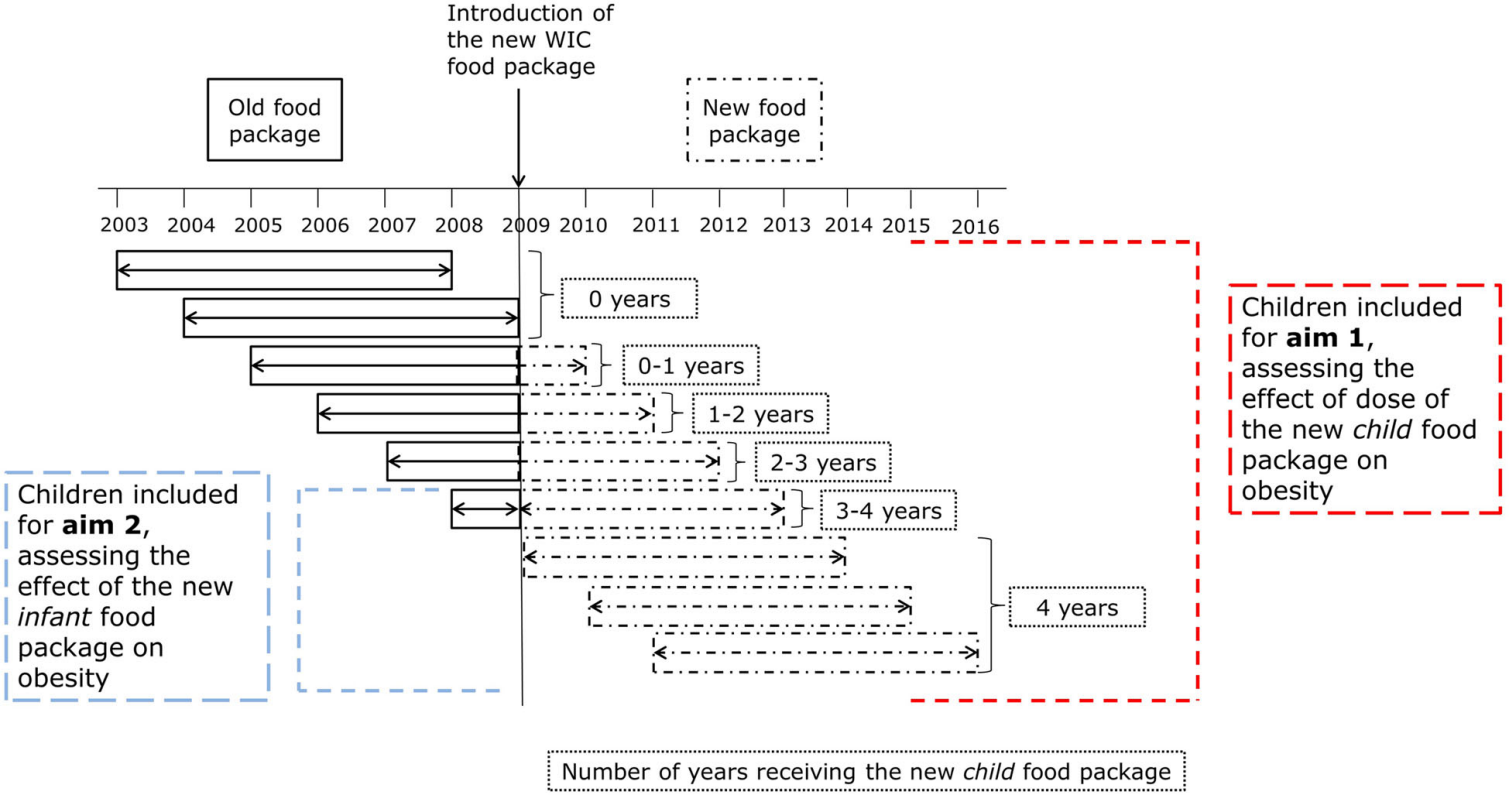
Groups of children who participated in WIC in Los Angeles County in 2003–2016 included in the study

Data came from the *Data Mining Project*, and included: 1) repeated measures of weight and recumbent length (or standing height) for all WIC-participating children in Los Angeles County at the time of certification (entry into the program) and re-certification (every 6–12 months); 2) sociodemographic information on children and their families; and 3) the type of food package children received from WIC throughout their participation. These data come from administrative records; weight and length (or height) are measured at each of the time points described while sociodemographic information is reported by the caregivers upon enrollment into WIC. Data on the type of food package provided by WIC is available for every month of participation. In this study we included children who: participated in WIC from 2003 until 2016 throughout ages 0–4 years (inclusive), enrolled in WIC within 42 days of birth, had at least one weight and length (height) measurement per year, and at least one measurement after the age of 4 years. We restricted the current analysis to children who received the *fully formula feeding* package every month during their first year of life; i.e., were never breastfed (*N* = 74,871). For aim 1, based on the calendar years in which the child participated in WIC (Fig. 1), we categorized children according to the duration of receipt (or dose) of the new *child* food package: 0 years, > 0 to < 1 years, 1 to < 2 years, 2 to < 3 years, 3 to < 4 years, and 4 years. Dose of the new child package was treated as a categorical variable.

Fully formula fed children participating in WIC pre-2009 received cumulatively more formula in the first year of life compared to children participating in WIC post-2009. Prior to the 2009 food package change, fully formula fed children received 403 fl oz. of formula each month during their first year of life, and 96 fl oz. of juice each month between 4 and 11.9 months. After the 2009 food package change, fully formula fed children received 403 fl oz. formula per month between 0 and 3.9 months, 442 fl oz. per month between 4 and 5.9 months, and 312 fl oz. per month between 6 and 11.9 months of age [3]. Juice was removed from the new food package which added a new benefit, 128 oz. of baby food fruits and vegetables monthly between 6 and 11.9 months.

To isolate the potential effect of the new *infant* food package on obesity for aim 2, we compared a sub-sample of children who received the old*infant* package followed by the new*child* package with those who received the new*infant* package and the new*child* package (Fig. 1). This analysis required comparison of the cohort born in the year before the food package change to children born in the three years following the food package change.

The outcomes of the study were weight-for-height z-score (WHZ) growth trajectories from birth until 4 years (inclusive) and obesity at age 4 years. WHZ values were estimated using sex-specific CDC growth curves [8]; subjects with implausible WHZ values (8 ≤ WHZ ≤ -4) were excluded from analyses [9]. WHZ was used to assess longitudinal growth trajectories because: 1) the CDC does not have body mass index (BMI)-for-age curves for children < 2 years of age, and 2) BMI is correlated with length (height) and thus may not be appropriate for assessing adiposity trajectories in growing children [10, 11]. Obesity at age 4 was defined as BMI-for-age ≥ 95th percentile. Covariates included children’s age, gender, and race/ethnicity as reported by their caregiver (Asian, African American, Hispanic, White, other); maternal education (less than high school, high school graduate, more than high school) and language preference (English, Spanish, other); and family income (< 50% federal poverty level [FPL], 50–100% FPL, > 100% FPL).

### Statistical analyses

Frequencies, means and standard deviations were calculated to characterize the sample. We assessed differences in growth trajectories by comparing mean WHZ slopes and differences in WHZ means between new child food package dosage groups (0 years, > 0 to < 1 years, 1 to < 2 years, 2 to < 3 years, 3 to < 4 years, and 4 years) in 6-month intervals for the first two years and each year thereafter (0–0.5, 0.5–1, 1–1.5, 1.5–2, 2–3, 3–4, and 4–5 years). Slopes were estimated using gender-stratified piecewise linear spline mixed models [12, 13], including a random intercept and random slopes for each age interval for each individual, as well as interactions between dose of the new WIC child package and age, allowing mean slope in each interval to vary by dose.

To adjust for confounding, we created post-stratification weights using child’s initial WHZ (categorized as − 4 to − 1, − 1 to 1, and 1 to 8); maternal education and language preference; and household income. Post-stratification weights were calculated as the ratio of the proportion of children in a joint covariate stratum in a reference population (i.e. children with an initial WHZ of greater than − 1 but less than 1, a mother with less than a high school education, a mother who prefers to speak Spanish, and a family income of < 50% FPL) to the proportion in that joint covariate stratum in each category of duration of the new child package received (0, 0 > and < 1, 1 to < 2, 2 to < 3, 3 to < 4 and 4 years). The reference population for this analysis was all children who received exclusively new infant and new child packages (i.e. first enrolled in WIC after October 1, 2009). Weights were calculated for strata defined by every combination of these covariates (initial WHZ; maternal education and language preference; and family income) in each category of the exposure (0, 0 > and < 1, 1 to < 2, 2 to < 3, 3 to < 4, and 4 years of the new child package). Race/ethnicity was excluded from post-stratification weights because of sparseness in joint covariate strata for non-Hispanic children, which created weight instability. In addition to incorporating these post-stratification weights, the mixed models were adjusted for maternal education and language preference, household income and child race/ethnicity.

We used gender-stratified modified Poisson regression models with robust standard error estimation [14] to assess the association between dose of the new child food package and obesity at age 4. Dose of the new WIC child package was treated as both a categorical and an interval-scaled variable. We used similar models to assess the association between *infant* food package type (old vs. new) and obesity among the sub-sample of children receiving > 3.5 years of the new *child* package. All Poisson models incorporated post-stratification weights and were adjusted for child race/ethnicity and initial WHZ; maternal education and language preference; and household income. Analyses were conducted using SAS 9.4 (SAS Institute Inc., Cary, NC). A *p*-value< 0.05 was considered to be of statistical significance.

## Results

Table 1 displays the characteristics of the sample, stratified by gender and by dose of the new child food package. The sample was predominately Hispanic (> 80%) across groups. Children receiving 4 years of the new child food package, compared to the other groups, had a higher proportion of English-speaking mothers, a smaller proportion of mothers with less than a high school education, and a larger proportion of severe family poverty (< 50% FPL). In addition, boys and girls receiving 4 years of the new child package had a lower WHZ at first measurement and lower obesity prevalence at age 4 compared to children in the other groups.

**Table 1:** Sample characteristics by gender and by dose of the new child package received^1^

	Number of years receiving the new *child* food package					
**BOYS**	**0** (***N*** **= 11,089**)	**> 0 to < 1** (***N*** **= 4361**)	**1 to < 2** (***N*** **= 4138**)	**2 to < 3** (***N*** **= 4436**)	**3 to < 4** (***N*** **= 4679**)	**4** (***N*** **= 9706**)
Initial WHZ, mean ± SD	0.49 ± 1.25	0.49 ± 1.20	0.55 ± 1.22	0.53 ± 1.23	0.53 ± 1.25	0.35 ± 1.25
Age (y) at initial WHZ, mean ± SD	0.29 ± 0.22	0.23 ± 0.23	0.25 ± 0.23	0.25 ± 0.24	0.26 ± 0.24	0.22 ± 0.23
Obese (BMI > 95th percentile) at 4y, N (%)	2795(25.4)	1165(26.9)	1094(26.7)	1134(25.8)	1166(25.1)	2232(23.1)
Child race/ethnicity, N (%)						
Asian	722(6.5)	223(5.1)	192(4.6)	186(4.2)	176(3.8)	381(3.9)
Black	741(6.7)	245(5.6)	226(5.5)	278(6.3)	328(7.0)	695(7.2)
Hispanic	9238(83.3)	3732(85.6)	3622(87.5)	3831(86.4)	4030(86.1)	8253(85.0)
White	376(3.4)	134(3.1)	82(2.0)	101(2.3)	102(2.2)	223(2.3)
Other	12(0.1)	27(0.6)	16(0.4)	40(0.9)	43(0.9)	154(1.6)
Parental language, N (%)						
English	4582(41.3)	1919(44.0)	1915(46.3)	2220(50.1)	2451(52.4)	5529(57.0)
Spanish	5977(53.9)	2261(51.9)	2089(50.5)	2096(47.3)	2103(45.0)	3921(40.4)
Other	530(4.8)	181(4.2)	134(3.2)	120(2.7)	125(2.7)	256(2.6)
Parental education, N (%)						
< high school	7081(63.9)	2756(63.2)	2645(63.9)	2780(62.7)	2813(60.1)	5288(54.5)
High school	3149(28.4)	1289(29.6)	1182(28.6)	1279(28.8)	1464(31.3)	3366(34.7)
> high school	859(7.8)	316(7.3)	311(7.5)	377(8.5)	402(8.6)	1052(10.8)
Family income, N (%)						
< 50% FPL	3314(29.9)	1234(28.3)	1131(27.3)	1262(28.5)	1324(28.3)	3472(35.8)
50–100% FPL	5093(45.9)	2139(49.1)	2013(48.7)	2077(46.8)	2162(46.2)	4405(45.4)
> 100% FPL	2682(24.2)	988(22.7)	994(24.0)	1097(24.7)	1193(25.5)	1829(18.8)
**GIRLS**	**0** (***N*** **= 10,552**)	**> 0 to < 1** (***N*** **= 4167**)	**1 to < 2** (***N*** **= 3979**)	**2 to < 3** (***N*** **= 4262**)	**3 to < 4** (***N*** **= 4508**)	**4** (***N*** **= 8994**)
Initial WHZ, mean ± SD	0.48 ± 1.19	0.49 ± 1.18	0.49 ± 1.16	0.49 ± 1.18	0.54 ± 1.14	0.37 ± 1.16
Age (y) at initial WHZ, mean ± SD	0.29 ± 0.22	0.24 ± 0.23	0.25 ± 0.23	0.25 ± 0.24	0.26 ± 0.24	0.22 ± 0.23
Obese (BMI > 95th percentile) at 4y, N (%)	2375(22.7)	938(22.7)	901(22.9)	958(22.7)	997(22.3)	1867(20.9)
Child race/ethnicity, N (%)						
Asian	673(6.4)	214(5.1)	173(4.4)	185(4.3)	179(4.0)	356(4.0)
Black	666(6.3)	273(6.6)	220(5.5)	275(6.5)	279(6.2)	683(7.6)
Hispanic	8820(83.6)	3556(85.3)	3472(87.3)	3680(86.3)	3910(86.7)	7602(84.5)
White	381(3.6)	109(2.6)	88(2.2)	101(2.4)	94(2.1)	199(2.2)
Other	12(0.1)	15(0.4)	26(0.7)	21(0.5)	46(1.0)	154(1.7)
Parental language, N (%)						
English	4401(41.7)	1890(45.4)	1891(47.5)	2053(48.2)	2378(52.8)	5141(57.2)
Spanish	5684(53.9)	2125(51.0)	1961(49.3)	2082(48.9)	2025(44.9)	3610(40.1)
Other	467(4.4)	152(3.7)	127(3.2)	127(3.0)	105(2.3)	243(2.7)
Parental education, N (%)						
< high school	6840(64.8)	2581(61.9)	2439(61.3)	2625(61.6)	2705(60.0)	4977(55.3)
High school	2851(27.0)	1275(30.6)	1239(31.1)	1330(31.2)	1415(31.4)	3175(35.3)
> high school	861(8.2)	311(7.5)	301(7.6)	307(7.2)	388(8.6)	842(9.4)
Family income, N (%)						
< 50% FPL	3207(30.4)	1243(29.8)	1124(28.3)	1157(27.2)	1297(28.8)	3308(36.8)
50–100% FPL	4746(45.0)	2009(48.2)	1857(46.7)	2033(47.7)	2088(46.3)	4061(45.2)
> 100% FPL	2599(24.6)	915(22.0)	998(25.1)	1072(25.2)	1123(24.9)	1625(18.1)

^1^The sample includes children who participated in the Special Supplemental Nutrition Program for Women, Infants and Children (WIC) continuously from birth until age 4 (inclusive) in Los Angeles County, California between 2003 and 2016 and who were exclusively formula fed during their first year of life

Abbreviations: *BMI* body mass index; *FPL* federal poverty level; *SD* standard deviation; *WHZ* weight-for-height z-score; y = years

**Fig. 2:**
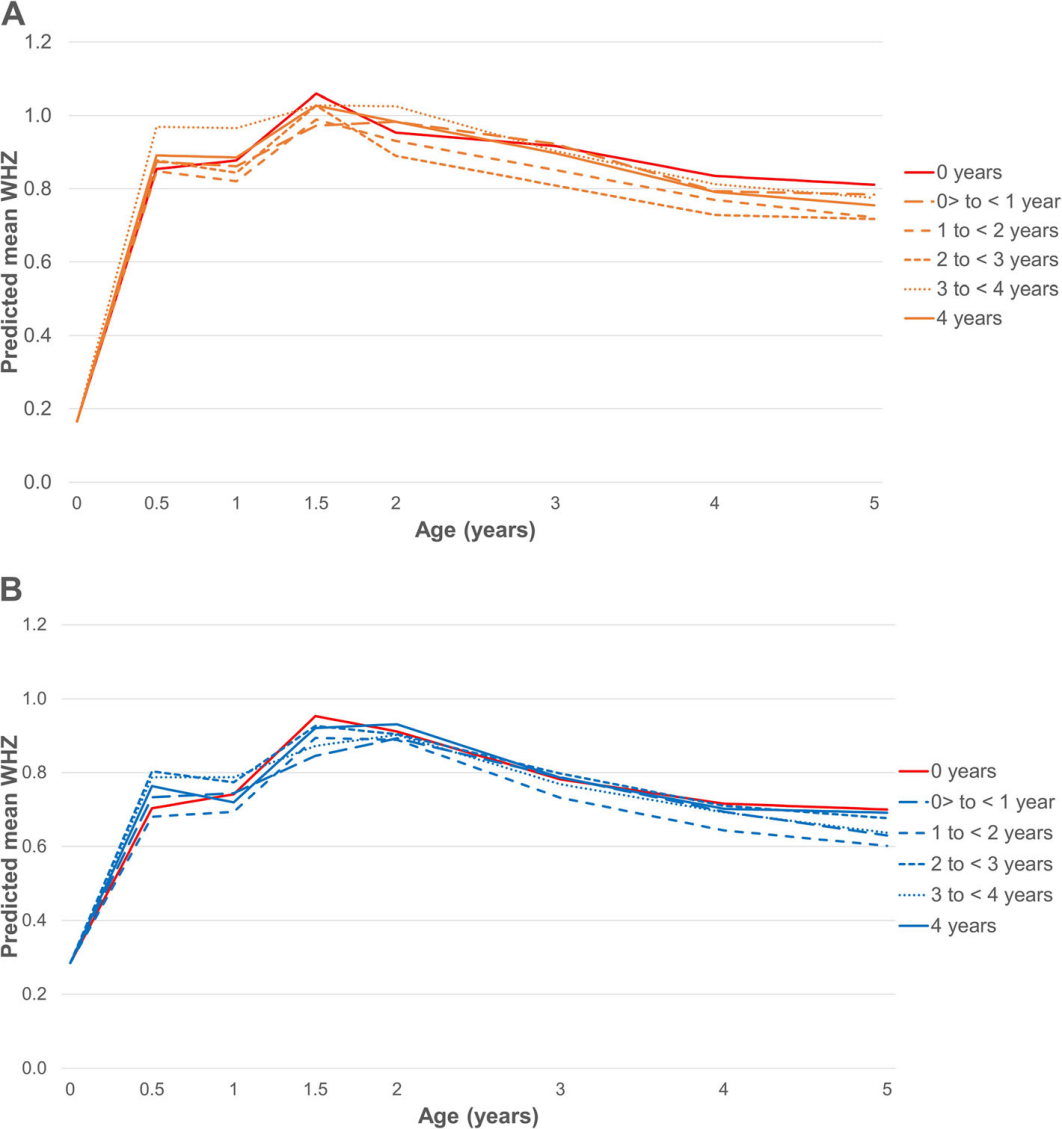
Growth trajectories for children 0–4 years who participated in WIC in Los Angeles County, 2003–2016. **a** Predicted WHZ means for boys by new child food package dose group. **b** Predicted WHZ means for girls by new child food package dose group

Figure 2 displays predicted WHZ means from age 0 to 5 by dosage of the new child food package for boys (Fig. 2a) and girls (Fig. 2b), with associated slope and mean WHZ differences by age and dose group displayed in Table 2. WHZ growth trajectories were similar for boys and girls across dose groups. For girls, the only significant differences in WHZ means appeared when comparing girls receiving 4 years of the new child food package vs. 0 years (Table 2), but the differences were small (less than 0.10 of a standard deviation in WHZ). For boys, there were significant differences in WHZ means in all dose comparisons at different ages, but these differences were also small.

**Table 2:** Spline mixed models investigating the association between new *child* package dose and WHZ growth trajectories^1^

	Slope differences (SE)			Mean WHZ differences (SE)		
Years of new *child* package received	Age (years)	Boys	Girls	Age (years)	Boys	Girls
< 1 year vs. 0 years	0 to 0.5	0.04 (0.10)	0.06 (0.10)	0	0.06 (0.03)	0.02 (0.03)
	0.5 to 1	− 0.07 (0.09)	− 0.05 (0.09)	0.5	0.08 (0.03)*	0.05 (0.03)
	1 to 1.5	−0.14 (0.08)	− 0.22 (0.08)**	1	0.04 (0.03)	0.03 (0.03)
	1.5 to 2	0.23 (0.06)***	0.18 (0.06)**	1.5	−0.03 (0.03)	−0.08 (0.03)
	to 3	−0.02 (0.02)	0.02 (0.02)	2	0.09 (0.03)***	0.00 (0.03)
	3 to 4	−0.05 (0.02)*	− 0.03 (0.02)	3	0.06 (0.02)**	0.03 (0.02)
	4 to 5	0.01 (0.04)	−0.05 (0.04)	4	0.02 (0.02)	0.00 (0.02)
				5	0.03 (0.04)	−0.05 (0.03)
1 to <2 years vs 0 years	0 to 0.5	−0.01 (0.10)	− 0.05 (0.10)	0	0.09 (0.03)**	0.04 (0.04)
	0.5 to 1	− 0.10 (0.09)	−0.05 (0.09)	0.5	0.09 (0.03)**	0.02 (0.03)
	1 to 1.5	−0.03 (0.08)	− 0.02 (0.08)	1	0.04 (0.03)	−0.01 (0.03)
	1.5 to 2	0.09 (0.06)	0.07 (0.06)	1.5	0.02 (0.03)	−0.02 (0.03)
	2 to 3	−0.04 (0.02)	−0.03 (0.02)	2	0.07 (0.03)**	0.02 (0.03)
	3 to 4	0.00 (0.02)	−0.02 (0.02)	3	0.03 (0.02)	−0.01 (0.02)
	4 to 5	−0.02 (0.04)	−0.03 (0.04)	4	0.03 (0.02)	−0.03 (0.02)
				5	0.00 (0.04)	−0.06 (0.03)
2 to < 3 years vs. 0 years	0 to 0.5	0.05 (0.10)	0.20 (0.10)	0	0.07 (0.03)*	−0.01 (0.03)
	0.5 to 1	−0.11 (0.09)	− 0.14 (0.09)	0.5	0.09 (0.03)**	0.09 (0.03)
	1 to 1.5	0.00 (0.08)	−0.12 (0.08)	1	0.04 (0.03)	0.02 (0.03)
	1.5 to 2	−0.06 (0.06)	0.04 (0.06)	1.5	0.04 (0.03)	−0.04 (0.03)
	2 to 3	−0.04 (0.02)	0.02 (0.02)	2	0.01 (0.03)	−0.02 (0.03)
	3 to 4	0.00 (0.02)	−0.02 (0.02)	3	−0.04 (0.02)	0.00 (0.02)
	4 to 5	0.01 (0.04)	−0.02 (0.04)	4	−0.04 (0.02)	−0.02 (0.02)
				5	−0.02 (0.04)	−0.04 (0.03)
3 to < 4 years vs. 0 years	0 to 0.5	0.23 (0.09)*	0.17 (0.09)	0	0.02 (0.03)	0.03 (0.03)
	0.5 to 1	−0.05 (0.08)	−0.08 (0.08)	0.5	0.13 (0.03)***	0.12 (0.03)
	1 to 1.5	−0.24 (0.08)**	−0.25 (0.07)***	1	0.11 (0.03)***	0.08 (0.03)
	1.5 to 2	0.21 (0.06)***	0.14 (0.06)*	1.5	−0.01 (0.03)	−0.05 (0.03)
	2 to 3	−0.09 (0.02)***	0.00 (0.02)	2	0.09 (0.03)	0.02 (0.03)
	3 to 4	−0.01 (0.02)	−0.01 (0.02)	3	0.01 (0.02)	0.02 (0.02)
	4 to 5	−0.01 (0.04)	−0.04 (0.04)	4	0.00 (0.02)	0.01 (0.02)
				5	−0.02 (0.03)	−0.03 (0.03)
4 years vs. 0 years	0 to 0.5	0.07 (0.08)	0.12 (0.08)	0	−0.05 (0.03)	−0.06 (0.03)*
	0.5 to 1	−0.06 (0.07)	−0.16 (0.07)	0.5	−0.01 (0.02)	0.00 (0.02)
	1 to 1.5	−0.08 (0.06)	−0.02 (0.06)	1	−0.04 (0.02)	− 0.08 (0.02)***
	1.5 to 2	0.12 (0.05)*	0.10 (0.05)*	1.5	−0.08 (0.02)***	−0.09 (0.02)***
	2 to 3	−0.05 (0.02)**	−0.02 (0.02)	2	−0.02 (0.02)	− 0.04 (0.02)*
	3 to 4	−0.02 (0.02)	−0.02 (0.02)	3	−0.06 (0.02)***	− 0.06 (0.02)**
	4 to 5	−0.01 (0.03)	0.01 (0.03)	4	−0.09 (0.02)***	−0.08 (0.02)***
				5	−0.10 (0.03)***	−0.07 (0.03)*

^1^Gender-stratified piecewise linear spline mixed models based on a sample of fully formula fed children 0–4 years participating in the Special Supplemental Nutrition Program for Women, Infants and Children (WIC) in Los Angeles County, California, 2003–2016

* < 0.05 ** < 0.01 *** < 0.001

Abbreviations: *SE* standard error; *WIC* Special Supplemental Nutrition Program for Women, Infants and Children; *WHZ* weight-for-height z-score

Despite being modest in magnitude, the observed differences in growth translated into a lower obesity risk for children receiving the new child package for 4 years. Fully formula fed boys and girls who received the new child food package for 4 years vs. those receiving the new child package for 0 years, had 7 and 6% lower risk of obesity, respectively (Table 3). No significant differences were found for children receiving < 4 years of the new child package vs. those receiving none, when dose was considered a categorical variable. When dose was operationalized as interval-scaled instead, we observed a significant reduction of obesity risk of 2% for boys and 1% for girls for each additional year of receipt of the new child package (Table 3). Among the sub-sample of formula fed children assessed for aim 2, there were no differences in obesity risk among those receiving the old vs. the new *infant* packages (Boys RR = 1.04, 95%CI 0.98–1.12; Girls RR = 1.00, 95%CI 0.93–1.08).

**Table 3:** Poisson regression investigating the association between new *child* package dose and obesity at 4 years^1^

	Boys (*N* = 38,409)		Girls (*N* = 36,462)	
Dosage of new *child* food package	RR	95% CI	RR	95% CI
**Categorical exposure**				
0 years (reference)	1.00		1.00	
< 1 year	1.05	0.99–1.12	1.00	0.94–1.07
1 to <2 years	1.02	0.96–1.09	0.98	0.92–1.05
2 to < 3 years	1.00	0.95–1.07	0.99	0.92–1.05
3 to < 4 years	0.98	0.92–1.04	0.96	0.89–1.02
4 years	**0.93**	**0.89–0.98**	**0.94**	**0.89–0.99**
**Interval-scaled exposure**	**0.98**	**0.98–0.99**	**0.99**	**0.98–1.00**

The 95% confidence interval for the bolded values does not include 1; this implies significance at the *p*<.05 level.

^1^Gender-stratified Poisson regression models based on a sample of fully formula fed children 0–4 years participating in the Special Supplemental Nutrition Program for Women, Infants and Children (WIC) in Los Angeles County, California, 2003–2016

Abbreviations: *CI* confidence interval; *RR* relative risk; *WIC* Special Supplemental Nutrition Program for Women, Infants and Children

## Discussion

This study focused on children who participated in WIC in Los Angeles County who were fully formula fed during infancy to investigate if changes to the *child* or the *infant* WIC food packages were associated with a previously reported lower obesity risk among formula fed, WIC-participating children receiving the new WIC package [7]. Chaparro et al. [7] reported that boys who were *fully formula fed* during their first year of life and received the old WIC food package (i.e., pre-2009) had a 13% higher risk of obesity than *fully formula fed* boys who received the new WIC food package (post-2009). In the current study, with a sample from the same WIC population but a different study design, we found that fully formula fed infants who received the new *child* package for 4 years had a 6–7% lower obesity risk at age 4 compared to fully formula fed infants receiving the old food package, whereas infants who received less than 4 years of the new package did not show a decreased obesity risk.

Previous studies among the WIC population have suggested that a longer breastfeeding duration is protective of childhood obesity [7, 15]; however, to our knowledge no previous studies have focused on evaluating the potential effect of the *child* food package; i.e., the food package that children receive between 1 and 4 years of age, on obesity. Research evaluating the impact of the new WIC food package has identified improvements in children’s diet quality post-food package change [16], including an increased consumption of fruits, vegetables, whole grains, and low-fat dairy [16] and a higher Healthy Eating Index [17]. On the other hand, evidence linking dietary changes in the preschool years to improved obesity outcomes is still scarce.

A recent systematic review evaluating interventions aimed at preventing childhood obesity identified only six studies focused on preschool settings, though their inclusion criteria were stringent, including only randomized controlled trials, quasi-experimental studies or natural experiments that included a control group and a minimum follow-up of 6 months for the preschool category [18]. Three out of the five studies based in the U.S. included a diet component, in addition to physical activity, and only one of these found a positive effect (i.e., lower adiposity outcomes for the intervention vs. the control group) [19]. Interestingly, though, this latter study by Natale et al. [19] was the only one that included a direct dietary intervention, changing the menus of childcare settings for low-income families in Miami, FL to include more healthy food and drink offerings; the other studies only addressed diet by providing nutrition education for parents [18]. In that sense, the Natale et al. [19] study may be somewhat comparable to our study as WIC provides healthy foods to participating children, in addition to nutrition education, and thus bypasses economic barriers related to purchasing such healthy foods.

We found no differences in obesity risk among fully formula fed children who received the old vs. the new *infant* package (with all receiving > 3.5 years of the new *child* package). This result suggests that the calibration of formula amounts to meet infants’ age and needs that was part of the WIC food package change, and/or the changes in food provided between 4 and 11.9 months (addition of baby foods fruits and vegetables, removal of juice), may not have had much impact on obesity risk for fully formula fed infants. While a previous study suggests that WIC food package assignments are a good proxy for infant feeding practices [20], the differences in the infant food packages for formula feeders before and after the package changes may have not been enough to translate into improved obesity outcomes. However, it is important to note that all the children included in this analysis were exposed to the new *child* food package for > 3.5 years, containing fruits, vegetables, whole grains, and low-fat dairy. The reduction in formula amounts and the inclusion of fruits and vegetables at 6–11.9 months may have benefitted children receiving the new food package when compared to those receiving the old in terms of adiposity during infancy, but it is possible these effects were “washed out” as children grew older given that they were all receiving healthier food offerings from ages 1–4 years. The growth curves presented in Fig. 2 lend some support to this hypothesis; comparing the 3–4 years vs. the 4 years dose groups, receiving the old*infant* package and the new*infant* package, respectively, we observe differences in WHZ in the 0–1 year period, with the former group having steeper WHZ growth trajectories compared to the latter.

While previous research suggests that breastfeeding is associated with a decreased obesity risk compared to formula feeding [21], only a few studies have investigated the association between amount of formula consumed and obesity risk among exclusively formula fed children. A study focused on WIC-participating children who were fully formula fed in Hawaii and Puerto Rico found no association between amount of formula consumed at 0–2 months and weight gain four months later [22]. Another WIC-focused study found that number of feeds per day was positively associated with weight gain between 6 and 12 months among fully formula fed infants. Similarly, Wood et al. [23] reported in a cluster randomized trial including four pediatric clinics in North Carolina that formula fed infants who consumed formula in large bottles (≥6 oz) at 2 months of age had greater weight gain by month 6 compared to infants using regular-sized bottles (< 6 oz).

### Strengths and limitations

This study is based on a large and well characterized sample of WIC-participating children in Los Angeles County, with data prospectively collected during WIC certification and re-certification visits. Children’s weight and length (or height) were measured by WIC staff, and these anthropometric measures have high validity [24]. We also had access to child, maternal, and family variables that could be considered confounders in the association between dose of the new child food package and obesity; these variables were used to estimate post-stratification weights to minimize confounding. However, we could not control for birth cohort since birth cohort determined exposure category (i.e. dosage of new child package); therefore, the observed differences could be attributable to birth cohort rather than package exposure. In addition, we did not have access to dietary intake variables for the children included in the study. Even though WIC food package assignments are a valid proxy for infant feeding practices [20], how close they align to dietary practices during childhood (1–4 years) is unknown. Moreover, we did not have information on children’s physical activity levels, which may be unbalanced between the child package dose groups and, thus, influence our results. Finally, this study included children who participated in WIC in Los Angeles County from birth until age 4 (inclusive) and who were fully formula fed as infants. Therefore, the generalizability of our results beyond other similar populations cannot be established.

## Conclusions

Among fully formula fed children participating in WIC in Los Angeles County from birth until age 4 years, receiving the new *child* WIC package for 4 years was associated with a 6–7% reduction in obesity risk, when compared to fully formula fed children receiving the old *child* WIC package. These results suggest that consuming the healthy food provided by WIC during early childhood (1–4 years), including fruits, vegetables, whole grains, and low-fat dairy, may be beneficial for obesity risk at age 4 among children who were never breastfed as infants. While reduction in obesity risk was modest in magnitude, the population health impact of the new *child* food package may still be substantial given that a sizeable proportion of WIC-participating children receive formula from WIC.

Breastfeeding is the gold-standard for infant feeding, providing health benefits for both mothers and infants [25]. As such, efforts within WIC to incentivize breastfeeding initiation and exclusivity should continue [26]. However, among children who are formula fed, the types of food provided by WIC may have an impact on obesity risk. Further research on the social and biological mechanisms underlying these relationships will provide further insights into the contribution of WIC benefits to child growth.

## Data Availability

The data used in this study comes from the *Data Mining Project* (https://apps.phfewic.org/Projects/DataMining.aspx). These data are not publicly available, and its use is restricted.

## Abbreviations

BMI: Body Mass Index
FPL: Federal poverty level
WHZ: Weight-for-height z-score
WIC: Special Supplemental Nutrition Program for Women, Infants and Children
